# Arthritis/Rheumatism and Depressive Symptoms in Middle-Aged and Older Chinese Adults: Regression and Symptom Network Evidence from CHARLS 2018

**DOI:** 10.3390/healthcare14152429

**Published:** 2026-08-06

**Authors:** Zhujun Chen, Xiang Duan, Hong Yang, Taimin Wu, Florian Hinterwimmer, Fahu Yuan, Yu Qiao, Xin Wang

**Affiliations:** 1 Department of Spine Surgery, Wuhan Fourth Hospital, Hubei Provincial Sports Medicine Center, Hubei Provincial Clinical Research Center for Orthopaedics, Hubei Key Laboratory of Sports Injury and Precision Therapy, Wuhan 430033, China; chenzhujun@whsdsyy.wecom.work (Z.C.); yanghong2@whdsyy.wecom.work (H.Y.); 2 Clinical Psychology and Psychotherapy, Institute of Psychology, Faculty of Psychology and Movement Sciences, University of Hamburg, 20146 Hamburg, Germany; xiang.duan@uni-hamburg.de; 3 Affiliated Wuhan Mental Health Center, Tongji Medical College of Huazhong University of Science and Technology, Wuhan 430012, China; tymon555@163.com; 4 Department of Orthopaedics and Sports Orthopaedics, School of Medicine and Health, TUM University Hospital, Technical University of Munich, 81675 Munich, Germany; florian.hinterwimmer@tum.de; 5 Institute for AI and Informatics in Medicine, School of Medicine and Health, TUM University Hospital, Technical University of Munich, 81675 Munich, Germany; 6 School of Medicine, Jianghan University, Wuhan 430056, China; yuanfh@jhun.edu.cn

**Keywords:** arthritis/rheumatism, depressive symptoms, symptom network analysis, middle-aged and older adults, CHARLS

## Abstract

**Background:** Arthritis and rheumatic diseases frequently co-occur with depressive symptoms in middle-aged and older adults. However, most previous studies have relied on total depression scores and have paid less attention to symptom-level associations. This study aimed to examine population-level and symptom-level associations between arthritis/rheumatism and depressive symptoms. **Methods:** Data were drawn from 15,721 adults aged ≥ 45 years from the 2018 wave of the China Health and Retirement Longitudinal Study (CHARLS), a nationally representative survey in China. Hierarchical linear regression analyses were used to examine the associations between arthritis/rheumatism and depressive symptoms. Network analysis was used to estimate symptom-level networks and compare depressive symptom networks between participants with and without arthritis/rheumatism. **Results:** After adjustment for sociodemographic and health-related covariates, arthritis/rheumatism remained significantly associated with higher depressive symptom levels (*β* = 0.07, *p* < 0.001). Network analysis showed that depressed mood and perceived effort were the most central symptoms, while restless sleep and perceived effort showed the strongest bridge connections with arthritis/rheumatism. Network comparison analyses indicated no significant differences in global network structure or global strength between participants with and without arthritis/rheumatism. However, four symptom connections were significantly stronger, and low hope showed reduced centrality in participants with arthritis/rheumatism. **Conclusions:** Arthritis/rheumatism was associated with greater depressive symptom burden and with specific symptom-level connections involving sleep disturbance and perceived effort. These findings highlight the potential value of screening for sleep problems and effort-related symptoms in adults with arthritis/rheumatism.

## 1. Introduction

Population aging has become a major demographic trend worldwide, posing substantial challenges to public health and social care systems [1]. Aging-related functional decline and chronic morbidity often emerge earlier, particularly from midlife onward. Adults aged 45 years and above experience a growing burden of multimorbidity and long-term disability [2,3]. Among this population, chronic non-communicable diseases are leading contributors to disease burden [4] and frequently co-occur with psychological distress, compromising well-being and quality of life [5]. Understanding patterns of physical-mental health comorbidity in mid- to late-life is therefore essential for promoting healthy and active aging.

Among chronic physical conditions, arthritis and rheumatic diseases are particularly prevalent in middle-aged and older adults. In China, population-based surveys have reported that the prevalence of rheumatic complaints ranges from 11.6% to 46.4% [6]. Arthritis and rheumatic diseases comprise a diverse group of conditions that primarily affect the joints and surrounding tissues and are typically associated with pain, functional limitations, and, in severe cases, substantial disability [7]. Beyond their physical burden, arthritis and rheumatic diseases are also associated with substantial psychological distress, particularly depressive symptoms [8].

Depression is one of the most common and disabling mental health conditions in later life, with a meta-analysis estimating an average prevalence of 31.74% among older adults [9]. Moreover, depression in middle-aged and older adults often follows a persistent course rather than representing a transient episode [10]. The co-occurrence of arthritis/rheumatism with depressive symptoms therefore represents an important clinical and public health concern. In a single-center retrospective analysis of routine rheumatology care, approximately 36–40% of patients with osteoarthritis or rheumatoid arthritis screened positive for anxiety, depression, and/or fibromyalgia [11]. A US population-based study similarly showed that adults with arthritis reported substantially higher rates of depression and severe psychological distress than those without arthritis [8].

Although prior research has documented an association between arthritis/rheumatism and depression, most studies have relied on total depression scores or diagnostic categories [8,9,10,11]. However, depression is clinically heterogeneous, and individuals with similar total scores may experience markedly different combinations of affective, cognitive, somatic, and interpersonal symptoms [12]. This distinction may be particularly relevant in arthritis/rheumatism, because specific depressive symptoms may be closely intertwined with the physical and functional burden of chronic illness [13,14]. Experimental evidence further showed that sleep loss exacerbated fatigue, depressive symptoms, and pain in patients with rheumatoid arthritis, highlighting the close interrelationships among these symptom domains [14].

A network analytic approach is particularly appropriate for examining arthritis–depression comorbidity. Network theory conceptualizes depressive symptoms as interacting components rather than interchangeable indicators of a single latent disorder, while network analysis estimates their conditional associations and can identify central symptoms and bridge symptoms linking arthritis/rheumatism with depressive symptoms [15,16,17]. To our knowledge, few studies have integrated population-level regression analysis with symptom-level network analysis to examine the association between arthritis/rheumatism and depressive symptoms within the same representative sample of middle-aged and older adults. Such an integrated approach may provide a more comprehensive understanding of both the overall association between arthritis/rheumatism and depressive symptoms and the specific symptom-level patterns underlying their co-occurrence.

The present study adopted a complementary analytic framework integrating epidemiological modeling and symptom-network analysis. Specifically, the study aimed to (a) quantify population-level associations between arthritis/rheumatism and depressive symptoms and (b) identify symptom-specific associations and key bridge symptoms linking arthritis/rheumatism to depressive symptoms. By integrating population and symptom analyses, the study aimed to provide a comprehensive yet methodologically transparent characterization of arthritis/rheumatism-depression comorbidity and deepen understanding of the intertwined physical-mental health burden in later life.

## 2. Methods

### 2.1. Data and Sample

For this cross-sectional study, data were obtained from the 2018 wave of the China Health and Retirement Longitudinal Study (CHARLS), the most recent available wave conducted under non-pandemic conditions and containing all consistently coded measures required for the analyses. CHARLS collected extensive sociodemographic, health, and psychological information through structured interviews conducted by trained investigators across 150 county-level units in China. Details of the sampling design and data quality control have been described elsewhere [18].

**Figure 1:**
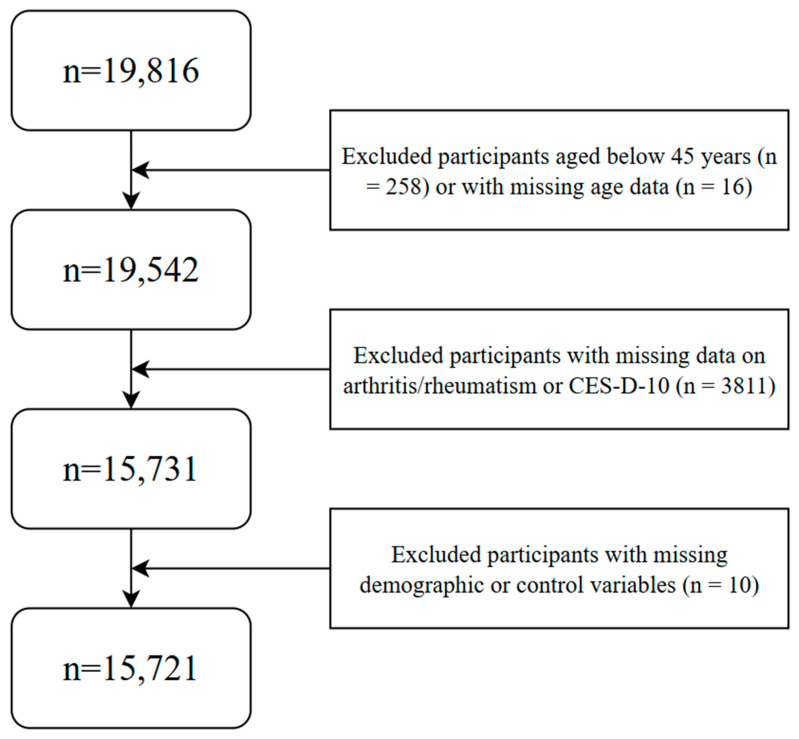
Flowchart of the sample selection process in the 2018 China Health and Retirement Longitudinal Study. CES-D-10: 10-item Center for Epidemiologic Studies Depression Scale.

The 2018 wave included 19,816 participants. Participants were included in the present analysis if they (1) were aged 45 years or older, (2) had complete data on arthritis/rheumatism and all depressive symptom items, and (3) provided valid responses for key sociodemographic and health-related variables. Individuals with missing information on any of these variables were excluded using complete-case analysis. The final analytical sample consisted of 15,721 respondents. A schematic overview of the participant inclusion process is presented in Figure 1.

In using the CHARLS data, we followed all ethical and legal requirements for secondary data analysis and strictly protected participant confidentiality. Formal authorization to access the database was obtained from the CHARLS research team prior to data download and analysis. The study procedures conformed to the ethical standards of the Declaration of Helsinki. The original CHARLS project received approval from the Biomedical Ethics Committee of Peking University (IRB00001052-11015), and all participants provided informed consent before participation.

### 2.2. Measures

#### 2.2.1. Arthritis/Rheumatism

Arthritis/rheumatism was assessed via participants’ self-reported physician diagnosis. Respondents were asked, “Have you been diagnosed with arthritis or rheumatism by a doctor?” Responses were coded as 0 = no and 1 = yes. The indicator served as the primary exposure variable. Because the CHARLS item did not distinguish between specific arthritis or rheumatic disease subtypes, it was treated as a broad indicator of physician-diagnosed arthritis/rheumatism.

#### 2.2.2. Depressive Symptoms

Depressive symptoms were measured using the 10-item Center for Epidemiologic Studies Depression Scale (CES-D-10) [19], which has been widely validated in older Chinese populations [20]. Participants rated the frequency of each symptom over the past week on a 4-point Likert scale (0 = “rarely or none of the time” to 3 = “most of the time”). Positive affect items (hopefulness and happiness) were reverse-coded, and higher total scores indicate more severe depressive symptoms. Internal consistency in the present study was good (Cronbach’s α = 0.81).

#### 2.2.3. Sociodemographic and Health-Related Variables

Sociodemographic covariates included age, sex, education, marital status, and residence. Age was analyzed in years. Sex was categorized as male or female. Education was classified as less than primary school, primary school, middle school, or high school and above. Marital status was categorized as married/cohabiting or unmarried/divorced/widowed, and residence was classified as rural or urban.

Health-related covariates included self-rated health, nighttime sleep duration, and the number of other non-psychiatric chronic conditions. Self-rated health was assessed using a single item asking participants to rate their general health as very good, good, fair, poor, or very poor. Nighttime sleep duration was assessed as the average number of hours of actual sleep per night during the previous month. Other non-psychiatric chronic conditions were identified from participants’ self-reported physician diagnoses of hypertension, dyslipidemia, diabetes or high blood sugar, cancer or malignant tumor, chronic lung disease, liver disease, heart disease, stroke, kidney disease, stomach or other digestive disease, memory-related disease, and asthma. The number of these conditions was summed to produce a non-psychiatric comorbidity count ranging from 0 to 12, with higher scores indicating a greater comorbidity burden.

Participants reporting physician-diagnosed emotional, nervous, or psychiatric problems were retained in the analytical sample, but this condition was excluded from the primary comorbidity count because of its conceptual overlap with depressive symptoms. General body pain severity, which was used only in an additional sensitivity analysis, was assessed using the item, “Are you often troubled with any body pains?” Response categories were none, a little, somewhat, quite a bit, or very.

### 2.3. Statistical Analyses

Descriptive statistics and hierarchical linear regression analyses were conducted using IBM SPSS Statistics for Windows, version 29.0 (IBM Corp., Armonk, NY, USA) [21] to examine sample characteristics and evaluate the association between arthritis/rheumatism and depressive symptoms.

Descriptive statistics were calculated for the total sample and separately for participants with and without arthritis/rheumatism. Group differences were examined using Welch’s independent-samples t tests for continuous variables and chi-square tests for categorical variables. Effect sizes were quantified using Cohen’s d and Cramér’s V, respectively.

Covariates were selected based on their potential confounding roles, conceptual relevance, and consistent availability in the 2018 CHARLS wave. In the hierarchical linear regression analysis, arthritis/rheumatism status was entered in Model 1. Sociodemographic covariates, including age, sex, education, marital status, and residence, were added in Model 2. Health-related covariates, including self-rated health, nighttime sleep duration, and the number of other non-psychiatric chronic conditions, were further entered in Model 3. Unstandardized regression coefficients (*B*), 95% confidence intervals (CIs), standardized regression coefficients (*β*), coefficients of determination (*R*^2^), and changes in explained variance (Δ*R*^2^) were reported. The statistical significance of changes in explained variance was evaluated using *F*-change tests. To examine whether the association between comorbidity burden and depressive symptoms differed by arthritis/rheumatism status, an interaction term between arthritis/rheumatism status and the mean-centered non-psychiatric comorbidity count was added to the fully adjusted model.

Regression assumptions and model influence were assessed using standardized residuals, residual-versus-fitted plots, quantile–quantile (Q–Q) plots, variance inflation factors, and Cook’s distance. HC3 heteroskedasticity-robust standard errors were used for statistical inference.

To assess potential selection bias associated with missing data, sociodemographic characteristics were compared between participants included in the final analytical sample and age-eligible participants excluded because of missing study variables. Welch’s independent-samples *t* test was used for age, and chi-square tests were used for categorical variables. Effect sizes were quantified using Cohen’s *d* and Cramér’s *V*, respectively. To account for multiple comparisons, *p* values were adjusted using the Benjamini–Hochberg false discovery rate procedure [22].

Sensitivity analyses were conducted to assess the robustness of the regression findings. First, the comorbidity count was recalculated after including self-reported physician-diagnosed emotional, nervous, or psychiatric problems. Second, the model was re-estimated without self-rated health and nighttime sleep duration to evaluate potential overadjustment for variables that may partly reflect downstream consequences of arthritis/rheumatism. Third, the fully adjusted model was additionally adjusted for general body pain severity, entered as a categorical variable with “none” as the reference category. Fourth, a missing-data sensitivity analysis was conducted using multiple imputation among all 19,542 age-eligible participants. Missing values in the exposure, outcome, and covariates were imputed across 20 datasets. The fully adjusted model was fitted in each dataset, and estimates were pooled using Rubin’s rules.

All network analyses were conducted in R 4.2.2 [23] with the packages mgm 1.2.15 [24], qgraph 1.9.8 [25], bootnet 1.8 [16], and NetworkComparisonTest 2.2.2 [26]. To examine the symptom-level conditional associations between arthritis/rheumatism and depressive symptoms, a mixed graphical model (MGM) was estimated in the total sample. The 10 depressive symptoms were modeled as Gaussian nodes, whereas arthritis/rheumatism status was modeled as a binary categorical node. Model estimation used the least absolute shrinkage and selection operator (LASSO), with 10-fold cross-validation used to select the optimal tuning parameter. The OR rule was used to symmetrize the nodewise estimates, such that an edge was retained if it was selected in at least one of the two corresponding nodewise regressions [24]. Expected influence (EI) was calculated to quantify the overall connectivity of each node, which can retain the direction of positive and negative edge weights [27]. Bridge expected influence (BEI) was calculated to identify depressive symptoms with the strongest connections to the arthritis/rheumatism node [17]. In addition, node predictability (*R*^2^) was estimated for each node to quantify the proportion of variance explained by its directly connected neighboring nodes [28].

To further explore whether the internal structure of depressive symptoms differed by arthritis/rheumatism, separate depressive symptom networks were estimated for participants with and without arthritis/rheumatism [26]. The Network Comparison Test (NCT) was used to examine group differences in (1) global strength, (2) overall network structure, (3) individual edge weights, and (4) expected influence using 1000 permutations, a commonly used setting that provides sufficiently stable permutation-based inference while maintaining computational feasibility [26]. To account for multiple testing, *p* values for individual edge and expected-influence comparisons were adjusted separately using the Benjamini–Hochberg false discovery rate procedure [22].

Network stability and accuracy were evaluated via nonparametric and case-dropping bootstraps (1000 resamples) using the bootnet package [16]. Nonparametric bootstrapping was used to estimate 95% confidence intervals for edge weights, whereas case-dropping bootstrapping was used to assess the stability of EI and BEI estimates. Stability was summarized using the correlation stability coefficient (CS coefficient). Following established guidelines, a CS coefficient greater than 0.25 indicates acceptable stability, whereas values above 0.50 are considered good [16].

## 3. Results

### 3.1. Descriptive Statistics

Participants were classified according to the presence or absence of self-reported physician-diagnosed arthritis/rheumatism. Of the 15,721 participants included in the final analysis, 9677 (61.6%) did not report arthritis/rheumatism and 6044 (38.4%) reported arthritis/rheumatism. As shown in Table 1, participants with arthritis/rheumatism were older, more frequently female, less educated, less frequently married or cohabiting, and more likely to live in rural areas than those without arthritis/rheumatism (all *p* < 0.001). Participants with arthritis/rheumatism also reported poorer self-rated health, shorter nighttime sleep duration, a greater number of other non-psychiatric chronic conditions, and higher depressive symptom severity (all *p* < 0.001). Among continuous variables, the largest standardized group differences were observed for comorbidity burden (Cohen’s *d* = 0.51) and depressive symptom severity (Cohen’s *d* = 0.47). Self-rated health was also moderately associated with arthritis/rheumatism status (Cramér’s *V* = 0.26).

The distribution of individual chronic conditions is presented in Supplementary Table S1. The most frequently reported conditions were hypertension (37.6%), stomach or other digestive disease (30.3%), dyslipidemia (22.7%), and heart disease (19.8%). Emotional, nervous, or psychiatric problems were reported by 2.6% of the analytical sample.

Compared with participants included in the final analysis, excluded age-eligible participants (*n* = 3821) were older (67.02 vs. 60.90 years, Cohen’s *d* = 0.62), more frequently female (57.5% vs. 51.2%, Cramér’s *V* = 0.05), less frequently married or cohabiting (74.7% vs. 87.5%, Cramér’s *V* = 0.14), and more likely to have less than primary-school education (59.7% vs. 35.4%, Cramér’s *V* = 0.20). A small difference was also observed in rural residence (61.4% vs. 59.3%, Cramér’s *V* = 0.02). All differences remained statistically significant after Benjamini–Hochberg false discovery rate correction. Detailed results are presented in Supplementary Table S2.

### 3.2. Hierarchical Linear Regression Analyses

The results of the hierarchical linear regression analyses predicting depressive symptom severity are presented in Table 2. In Model 1, participants with arthritis/rheumatism scored 2.96 points higher in depressive symptom severity than those without arthritis/rheumatism (*B* = 2.96, 95% CI 2.75–3.17, *β* = 0.22, *p* < 0.001). The model explained 4.9% of the variance in depressive symptom severity (*R*^2^ = 0.049). After age, sex, education, marital status, and residence were added in Model 2, the association remained significant (*B* = 2.40, 95% CI 2.19–2.61, *β* = 0.18, *p* < 0.001). Model 2 explained 10.9% of the variance (*R*^2^ = 0.109, Δ*R*^2^ = 0.060).

After self-rated health, nighttime sleep duration, and the number of other non-psychiatric chronic conditions were added in Model 3, the association between arthritis/rheumatism and depressive symptom severity was attenuated but remained significant (*B* = 0.99, 95% CI 0.80–1.19, *β* = 0.07, *p* < 0.001). The fully adjusted model explained 26.4% of the variance in depressive symptom severity (*R*^2^ = 0.264, Δ*R*^2^ = 0.155). Poorer self-rated health, shorter nighttime sleep duration, and a greater comorbidity count were independently associated with greater depressive symptom severity. Each additional non-psychiatric chronic condition was associated with a 0.31-point increase in depressive symptom severity (*B* = 0.31, 95% CI 0.25–0.38, *β* = 0.08, *p* < 0.001). The arthritis/rheumatism × comorbidity-count interaction was not significant (*B* = 0.10, 95% CI −0.02–0.21, *p* = 0.098).

The results remained robust across sensitivity analyses. When emotional, nervous, or psychiatric problems were additionally included in the comorbidity count, the association between arthritis/rheumatism and depressive symptom severity remained comparable to that in the primary analysis (*B* = 0.98, 95% CI 0.79–1.18, *p* < 0.001). In the reduced-adjustment model excluding self-rated health and nighttime sleep duration, the association was stronger but remained in the same direction (*B* = 1.75, 95% CI 1.54–1.95, *p* < 0.001). After additional adjustment for general body pain severity, the association between arthritis/rheumatism and depressive symptom severity was attenuated but remained significant (*B* = 0.54, 95% CI 0.35–0.74, *β* = 0.04, *p* < 0.001). Multiple imputation among all 19,542 age-eligible respondents also yielded a similar estimate (*B* = 0.93, 95% CI 0.75–1.10, *p* < 0.001), supporting the robustness of the complete-case findings. The sensitivity and interaction analyses are summarized in Supplementary Table S3.

No serious multicollinearity was observed, with variance inflation factors ranging from 1.06 to 2.87. Residual diagnostics indicated heteroskedasticity; therefore, HC3 heteroskedasticity-robust standard errors were used for statistical inference. The maximum Cook’s distance was 0.002, indicating no influential individual observations.

### 3.3. Network Analysis of Arthritis/Rheumatism and Depressive Symptoms

**Figure 2:**
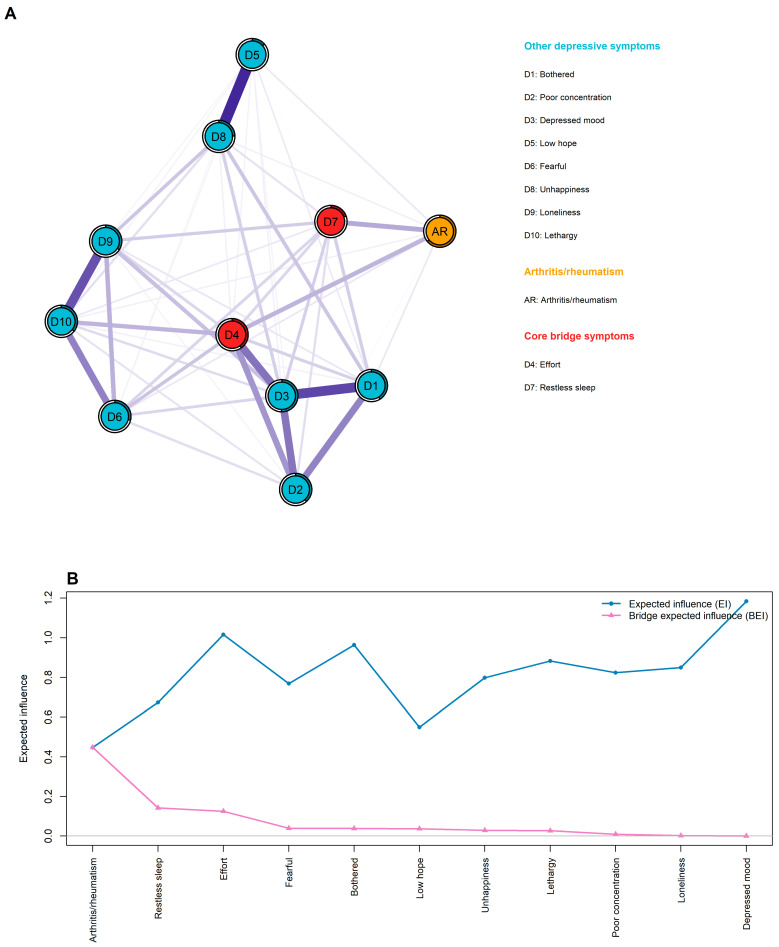
Network structure and centrality indices of arthritis/rheumatism and depressive symptoms. (**A**) Estimated network structure. Nodes represent arthritis/rheumatism and depressive symptoms. Blue nodes indicate depressive symptoms, the yellow node represents arthritis/rheumatism, and red nodes indicate the two depressive symptoms with the highest BEI values. Edges represent regularized conditional associations, and thicker edges indicate stronger associations. Rings represent predictability. (**B**) Centrality indices. EI and BEI are displayed for all nodes, ordered by BEI. EI reflects the overall connectivity of a node within the network, whereas BEI reflects the extent to which a node connects the depressive symptom community with the arthritis/rheumatism node.

The estimated network structure (Figure 2A) and the centrality indices, including BEI and EI (Figure 2B), are presented for arthritis/rheumatism and depressive symptoms. Edge weights ranged from 0.01 to 0.35, reflecting small-to-moderate regularized conditional associations between nodes.

The arthritis/rheumatism node had a BEI value of 0.45. Among depressive symptoms, D7 (restless sleep, BEI = 0.14) and D4 (effort, BEI = 0.13) had the highest BEI values. D3 (depressed mood, EI = 1.18) and D4 (effort, EI = 1.02) exhibited the highest EI, followed by D1 (bothered) and D10 (lethargy) (Table 3). Node predictability (R^2^) for depressive symptoms ranged from 0.14 to 0.45, with a mean of 0.33, indicating that roughly one-third of the variance in depressive symptoms could be explained by their neighboring nodes.

Case-dropping bootstrap results indicated good stability of the EI and BEI estimates (Figure S1). The CS coefficients were 0.75 for both EI and BEI, indicating good stability; up to 75% of cases could be dropped while retaining, with 95% probability, a correlation of at least 0.70 between the subset and original centrality estimates [16]. The nonparametric bootstrap confidence intervals suggested acceptable accuracy of the estimated edge weights.

### 3.4. Comparative Network Analysis of Depressive Symptoms in Participants with and Without Arthritis/Rheumatism

**Figure 3:**
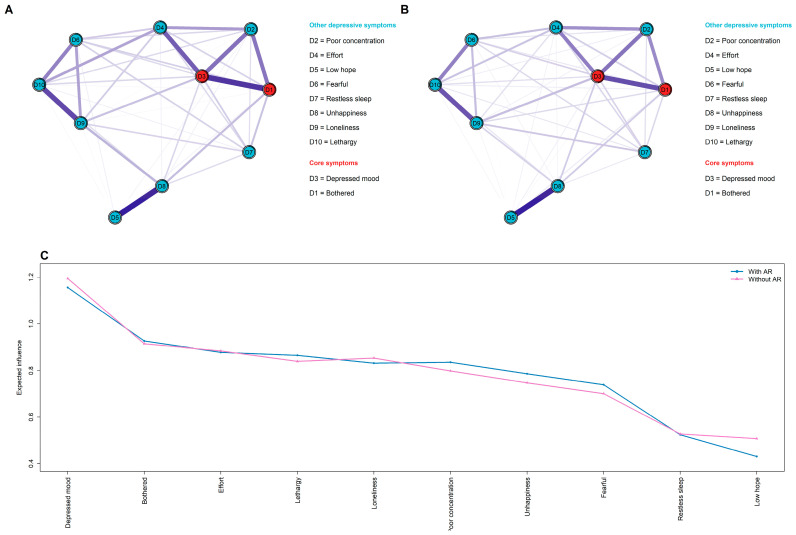
Comparison of depressive symptom network structure and expected influence between participants with and without arthritis/rheumatism**.** (**A**) Depressive symptom network among participants with arthritis/rheumatism. (**B**) Depressive symptom network among participants without arthritis/rheumatism. (**C**) Comparison of expected influence between the two groups. Edge thickness reflects the strength of the association and colors represent the direction of associations (purple = positive; red = negative). Rings represent predictability. AR = arthritis/rheumatism.

To further examine whether depressive symptom structures differed by arthritis/rheumatism, Figure 3 presents the estimated symptom networks and EI values for participants with and without arthritis/rheumatism. In the arthritis/rheumatism group, D3 (depressed mood) showed the highest EI (1.16), followed by D1 (bothered; EI = 0.93). A highly similar pattern was observed among participants without arthritis/rheumatism, where D3 (depressed mood) also exhibited the highest EI (1.20), followed by D1 (bothered; EI = 0.92). The stability of EI estimates was high in both groups (CS = 0.75), indicating robust estimation of EI (Figure S2). Overall, the ranking and magnitude of central nodes were largely consistent across groups.

The Network Comparison Test (NCT) showed no significant between-group difference in overall network structure (*M* = 0.05, *p* = 0.32) or global strength (*S* = 0.09, *p* = 0.46). After adjustment for multiple comparisons, four individual edges differed significantly between the two networks: D2–D4 (poor concentration-effort, E = 0.03, FDR-adjusted *p* = 0.049), D8–D9 (unhappiness-loneliness, E = 0.04, FDR-adjusted *p* = 0.03), D5–D8 (low hope-unhappiness, E = 0.04, FDR-adjusted *p* = 0.02), and D1–D9 (bothered-loneliness, E = 0.05, FDR-adjusted *p* = 0.02). All four edges were stronger in the arthritis/rheumatism group. Centrality invariance tests further showed that D5 (low hope) had lower EI in the arthritis/rheumatism group (Δ = −0.10, FDR-adjusted *p* = 0.02).

## 4. Discussion

Using nationally representative data from middle-aged and older adults in China, our study employed hierarchical linear regression and symptom network analysis to examine the relationship between arthritis/rheumatism and depressive symptoms. Arthritis/rheumatism was significantly associated with higher depressive symptom levels after adjustment for sociodemographic and health-related variables, while the depressive symptom network was organized around the core nodes “depressed mood” and “everything was an effort”. “Restless sleep” and “everything was an effort” emerged as key bridge symptoms linking arthritis/rheumatism with depressive symptoms. Importantly, four specific symptom connections were significantly stronger in the arthritis/rheumatism group, alongside a reduction in the centrality of low hope. These findings suggested that arthritis/rheumatism may not be associated with a fundamental reorganization of the depressive symptom network but may instead be associated with stronger local symptom interactions and reduced centrality of low hope.

### 4.1. Arthritis/Rheumatism Status and Depressive Symptoms

In the analytical sample, 6044 participants (38.4%) reported a physician diagnosis of arthritis/rheumatism. Previous Chinese epidemiological studies have reported considerable variation in the prevalence of rheumatic complaints according to case definition, assessment method, age, and geographical location [6]. Individuals with arthritis/rheumatism had greater depressive symptom severity than those without arthritis/rheumatism. This finding is consistent with previous population-based evidence of poorer psychological well-being among adults with arthritis [8].

Hierarchical linear regression showed that the arthritis/rheumatism-depressive symptoms association remained significant after adjusting for sociodemographic characteristics, self-rated health, nighttime sleep duration, and non-psychiatric comorbidity burden. A greater number of other non-psychiatric chronic conditions was also independently associated with greater depressive symptom severity. Previous national evidence has similarly linked chronic diseases and multimorbidity to poorer psychological health and depression [29,30]. The association between arthritis/rheumatism and depressive symptoms remained robust across all sensitivity analyses, including alternative specifications of the comorbidity count, reduced covariate adjustment, additional adjustment for general body pain severity, and multiple imputation, while the arthritis/rheumatism × comorbidity count interaction was not significant, indicating no clear evidence that the association between comorbidity burden and depressive symptom severity differed by arthritis/rheumatism status. Comparable estimates from the multiple-imputation and alternative-comorbidity analyses further supported the robustness of the primary regression findings. Overall, the results indicate a modest cross-sectional association between arthritis/rheumatism and depressive symptom severity in middle-aged and older adults.

### 4.2. Symptom Network of Arthritis/Rheumatism and Depressive Symptoms

The strong bridge connections of D7 (restless sleep) and D4 (effort) with arthritis/rheumatism are particularly noteworthy. Bridge expected influence identifies symptoms with relatively strong cross-community conditional associations. In the present network, D7 and D4 showed the strongest connections between the depressive symptoms and the arthritis/rheumatism node. The bridge role of restless sleep is broadly consistent with network research identifying sleep disturbance as a central or bridging symptom across depression and anxiety [31]. One possible explanation is that nocturnal awakenings and impaired sleep continuity associated with arthritis/rheumatism may intensify physical discomfort and depressive distress [14]. Sleep disturbance interacts with lethargy and somatic complaints [32], while temporal studies show that sleep fluctuations predict next-day stress and affective deterioration [33]. The item “everything was an effort” may be particularly relevant among individuals with arthritis/rheumatism because it can overlap with the somatic and functional burden accompanying arthritis [34]. Network analyses in older adults similarly show that “inability to get going” and “everything was an effort” occupy central or bridge positions linking depressive symptoms with quality of life and physical functioning [35]. In arthritis/rheumatism, routine activities such as walking, climbing stairs, or performing household tasks become increasingly demanding due to pain and joint stiffness [7]. Thus, “everything was an effort” likely reflects the subjective integration of reduced physical capacity and motivational depletion and may function as a pathway linking physical limitations with depressive symptoms.

In addition to the bridge symptoms, the high centrality of D3 (depressed mood) and D4 (effort) indicated that these symptoms constitute the core of the depressive symptom structure in middle-aged and older adults. Such a pattern has also been observed in community and clinical network studies showing that depressed mood and effort-like symptoms are core drivers of late-life depression [35,36].

### 4.3. Comparative Depressive Symptom Networks in Participants with and Without Arthritis/Rheumatism

We found no significant differences in overall network structure or global strength between participants with and without arthritis/rheumatism, consistent with prior network research showing that group differences often emerge in specific pathways rather than in whole-network architecture [17,26]. In chronic illness populations, etiological or psychosocial stressors frequently alter local symptom dynamics without producing large-scale topological shifts, particularly when the depressive phenotype is relatively homogeneous [37]. Additionally, individuals with and without arthritis/rheumatism often share age-related risk factors, such as multimorbidity, reduced mobility, and social role changes, which may further minimize global-level differences [38,39].

Despite the absence of global differences, several edge-specific differences emerged: connections between D2–D4 (poor concentration-effort), D8–D9 (unhappiness-loneliness), D5–D8 (low hope-unhappiness), and D1–D9 (bothered-loneliness) were significantly stronger among participants with arthritis/rheumatism. Stronger D2–D4 coupling aligns with evidence suggesting that chronic pain and activity limitation may increase cognitive load and reduce attentional resources [40], making effort feel more taxing for individuals with arthritis/rheumatism. Enhanced D8–D9 and D1–D9 associations indicate that mobility restrictions reduce opportunities for meaningful social participation and increase perceived social isolation [41,42]. The stronger D5–D8 pathway may similarly reflect the impact of functional uncertainty on positive affective experiences [43]. Collectively, these stronger associations may reflect differences in emotional, motivational, and social symptom interrelationships among participants with arthritis/rheumatism.

Centrality invariance tests showed that D5 (low hope) had significantly lower expected influence among participants with arthritis/rheumatism. This finding suggests that low hope may play a less integrated role in the broader depressive symptom network among individuals with arthritis/rheumatism. Together with stronger associations involving unhappiness, loneliness, and feeling bothered, this pattern may suggest a greater relative prominence of interpersonal and negative-affect symptoms among participants with arthritis/rheumatism. However, this interpretation should be treated with caution, and further longitudinal and experimental studies are needed to clarify the role of low hope in the dynamic interplay of depressive symptoms in populations with arthritis/rheumatism.

### 4.4. Limitations and Implications

The study has several limitations. First, the cross-sectional design precludes causal inference regarding the directionality of associations between arthritis/rheumatism and depressive symptoms. Although network analysis captures conditional dependencies among symptoms, it does not establish temporal or causal relationships, and the observed associations may partly reflect unmeasured common factors. Longitudinal or intensive repeated-measure designs are needed to clarify the temporal ordering and within-person dynamics of arthritis/rheumatism and depressive symptoms. Second, arthritis/rheumatism was assessed using a self-reported physician diagnosis, which may be subject to recall or reporting error and does not distinguish among osteoarthritis, rheumatoid arthritis, and other rheumatic conditions. The heterogeneity of this broad exposure may obscure subtype-specific associations with depressive symptoms. Future studies using clinically verified subtype-specific diagnoses are warranted. Third, several relevant socioeconomic, behavioral, and disease-related factors were unavailable or not included, including income, body mass index, physical activity, and detailed clinical information, which may have resulted in residual confounding. Although general body pain was considered in a sensitivity analysis, the available measure was nonspecific. Fourth, comorbidity was assessed using an unweighted count of chronic conditions, which did not account for differences in the severity or clinical impact of individual diseases and may therefore have incompletely captured overall comorbidity burden. Fifth, excluded age-eligible participants differed from included participants, particularly in age and education, indicating potential selection bias. However, the comparable multiple-imputation results partly mitigate this concern. Sixth, depressive symptoms were measured using the CES-D-10, a brief self-report instrument. While widely validated, it does not establish a clinical diagnosis of depression and may not capture the full complexity of depressive symptomatology. In addition, reliance on self-reported information may have introduced reporting bias.

Despite these limitations, several implications should be considered. First, the study provides population-level evidence that arthritis/rheumatism is associated with elevated depressive symptoms after adjustment for the measured sociodemographic and health-related covariates. Additionally, public health and clinical screening efforts may give particular attention to older adults with arthritis/rheumatism who also have sociodemographic characteristics associated with greater depressive symptom burden, such as female sex and lower educational attainment. These findings support consideration of depressive symptom screening among middle-aged and older adults reporting arthritis/rheumatism, particularly when sleep disturbance or effort-related symptoms are present. Second, by adopting a symptom network approach, the study identified specific conditional associations between arthritis/rheumatism and depressive symptoms. In particular, restless sleep and perceived effort showed the strongest bridge connections with the arthritis/rheumatism node, highlighting these symptoms as potentially relevant for further assessment and prospective research. Third, several small but significant differences were observed in specific edges and in the expected influence of low hope after multiple-testing correction. These findings suggest that future studies may benefit from examining localized symptom associations in addition to global network metrics.

## 5. Conclusions

Using a nationally representative sample and a combined regression and symptom-network approach, our study found that self-reported physician-diagnosed arthritis/rheumatism was significantly associated with higher depressive symptom levels in middle-aged and older Chinese adults. At the symptom level, depressed mood and “everything was an effort” showed the strongest expected influence, while “restless sleep” and “everything was an effort” showed the strongest bridge connections between the arthritis/rheumatism node and the depressive symptom community. Several localized differences were observed in specific symptom connections and in the expected influence of low hope, although their clinical significance remains uncertain. Clinically, the results highlight the potential value of assessing sleep disturbance and perceived effort among individuals with arthritis/rheumatism who report depressive symptoms. Brief psychological assessment may help identify co-occurring depressive symptoms in routine care. However, longitudinal studies are needed to clarify the direction of these associations, confirm the stability of the network findings, and determine whether sleep disturbance or perceived effort represent useful intervention targets.

## Figures and Tables

**Table 1:** Characteristics of the analytical sample by arthritis/rheumatism status.

Characteristic	Category	Total Sample (*n* = 15,721)	Without Arthritis/Rheumatism (*n* = 9677)	With Arthritis/Rheumatism (*n* = 6044)	*p*	Effect Size
Age		60.90 ± 9.32	59.97 ± 9.37	62.39 ± 9.06	<0.001	*d* = 0.26
Sex	Male	7678 (48.8%)	5145 (53.2%)	2533 (41.9%)	<0.001	*V* = 0.11
	Female	8043 (51.2%)	4532 (46.8%)	3511 (58.1%)		
Education	Less than primary school	5573 (35.4%)	2924 (30.2%)	2649 (43.8%)	<0.001	*V* = 0.15
	Primary school	4935 (31.4%)	3220 (33.3%)	1715 (28.4%)		
	Middle school	3260 (20.7%)	2119 (21.9%)	1141 (18.9%)		
	High school and above	1953 (12.4%)	1414 (14.6%)	539 (8.9%)		
Marital status	Married/cohabiting	13,763 (87.5%)	8607 (88.9%)	5156 (85.3%)	<0.001	*V* = 0.05
	Unmarried/divorced/widowed	1958 (12.5%)	1070 (11.1%)	888 (14.7%)		
Residence	Rural	9319 (59.3%)	5427 (56.1%)	3892 (64.4%)	<0.001	*V* = 0.08
	Urban	6402 (40.7%)	4250 (43.9%)	2152 (35.6%)		
Self-rated health	Very good	2018 (12.8%)	1624 (16.8%)	394 (6.5%)	<0.001	*V* = 0.26
	Good	2051 (13.0%)	1549 (16.0%)	502 (8.3%)		
	Fair	7810 (49.7%)	4843 (50.0%)	2967 (49.1%)		
	Poor	2958 (18.8%)	1316 (13.6%)	1642 (27.2%)		
	Very poor	884 (5.6%)	345 (3.6%)	539 (8.9%)		
Nighttime sleep duration (hours)		6.23 ± 1.90	6.43 ± 1.78	5.92 ± 2.03	<0.001	*d* = − 0.27
Other non-psychiatric chronic conditions (count)		1.73 ± 1.69	1.41 ± 1.49	2.25 ± 1.85	<0.001	*d* = 0.51
Depressive symptom score		8.43 ± 6.49	7.29 ± 5.95	10.25 ± 6.88	<0.001	*d* = 0.47

*Note.* Values are presented as mean ± standard deviation or *n* (%). Continuous variables were compared using Welch’s t tests, and categorical variables were compared using chi-square tests. Effect sizes are reported as Cohen’s *d* for continuous variables and Cramér’s *V* for categorical variables. Positive Cohen’s *d* values indicate higher means in participants with arthritis/rheumatism, whereas negative values indicate lower means.

**Table 2:** Hierarchical linear regression predicting depressive symptoms.

Predictor	Category	Model 1 B (95% CI)	Model 1 *β*	Model 2 B (95% CI)	Model 2 *β*	Model 3 B (95% CI)	Model 3 *β*
Arthritis/rheumatism	With arthritis/rheumatism	2.96 (2.75–3.17)	0.22 ***	2.40 (2.19–2.61)	0.18 ***	0.99 (0.80–1.19)	0.07 ***
Age	Per one-year increase			−0.003 (−0.014–0.008)	−0.004	−0.040 (−0.050–−0.029)	−0.06 ***
Sex	Female			1.49 (1.29–1.69)	0.12 ***	1.18 (0.99–1.36)	0.09 ***
Education	Less than primary school			2.19 (1.87–2.50)	0.16 ***	1.88 (1.59–2.17)	0.14 ***
	Primary school			1.00 (0.70–1.29)	0.07 ***	0.88 (0.61–1.15)	0.06 ***
	Middle school			0.73 (0.43–1.04)	0.05 ***	0.57 (0.29–0.85)	0.04 ***
Marital status	Unmarried/divorced/widowed			1.58 (1.25–1.92)	0.08 ***	1.32 (1.02–1.62)	0.07 ***
Residence	Rural			1.42 (1.22–1.62)	0.11 ***	1.22 (1.04–1.41)	0.09 ***
Self-rated health	Good					0.86 (0.57–1.15)	0.04 ***
	Fair					2.20 (1.95–2.44)	0.17 ***
	Poor					5.24 (4.90–5.59)	0.32 ***
	Very poor					7.41 (6.88–7.94)	0.26 ***
Nighttime sleep duration	Per one-hour increase					−0.59 (−0.65–−0.54)	−0.17 ***
Other non-psychiatric chronic conditions	Per one-condition increase					0.31 (0.25–0.38)	0.08 ***
R^2^		0.049		0.109		0.264	
Adjusted R^2^		0.049		0.108		0.264	
ΔR^2^				0.060 ***		0.155 ***	

*Note.* B = unstandardized regression coefficient; *β* = standardized regression coefficient; CI = confidence interval. HC3 heteroskedasticity-robust standard errors were used. Reference groups were participants without arthritis/rheumatism, male participants, those with high school education or above, married/cohabiting participants, urban residents, and participants reporting very good self-rated health. For regression coefficients, significance was based on coefficient tests; for Δ*R*^2^, significance was based on F-change tests. *** *p* < 0.001.

**Table 3:** EI and BEI of arthritis/rheumatism and depressive symptoms.

Node	Variables	EI	BEI
AR	Arthritis/rheumatism	0.45	0.45
D7	Restless sleep	0.67	0.14
D4	Effort	1.02	0.13
D6	Fearful	0.77	0.04
D1	Bothered	0.96	0.04
D5	Low hope	0.55	0.04
D8	Unhappiness	0.80	0.03
D10	Lethargy	0.88	0.03
D2	Poor concentration	0.82	0.01
D9	Loneliness	0.85	0.002
D3	Depressed mood	1.18	0.00

*Note.* EI = expected influence; BEI = bridge expected influence. Nodes are ordered by BEI. Higher EI values indicate greater overall connectivity of a node within the network, whereas higher BEI values indicate stronger connections between the depressive symptom community and the arthritis/rheumatism node.

## Data Availability

Publicly available datasets were analyzed in this study. The data used in this study are available from the China Health and Retirement Longitudinal Study (CHARLS: http://charls.pku.edu.cn/) upon registration and application through its official website. The authors do not have permission to redistribute the data.

## Supplementary Materials

The following supporting information can be downloaded at: https://www.mdpi.com/article/10.3390/healthcare14152429/s1, Table S1. Distribution of self-reported physician-diagnosed chronic conditions in the analytical sample; Table S2. Comparison of sociodemographic characteristics between included and excluded age-eligible participants; Table S3. Sensitivity and interaction analyses for the association between arthritis/rheumatism and depressive symptoms; Figure S1. Bootstrap accuracy and stability of the arthritis/rheumatism–depressive symptom network; Figure S2. Case-dropping bootstrap stability of expected influence in participants with and without arthritis/rheumatism.
