# Supplementary material for: Arthritis/Rheumatism and Depressive Symptoms in Middle-Aged and Older Chinese Adults: Regression and Symptom Network Evidence from CHARLS 2018
Source: Healthcare (Basel). 2026 Aug 6;14(15):2429. doi: 10.3390/healthcare14152429 (PMC13465139; doi:10.3390/healthcare14152429)
Supplement: Supplementary file 1 [file healthcare-14-02429-s001.zip › healthcare-4412456-supplementary.pdf]

Table S1

Distribution of self-reported physician-diagnosed chronic conditions in the analytical sample.

Chronic condition	Total sample (n = 15721)	Without arthritis/rheumatism (n = 9677)	With arthritis/rheumatism (n = 6044)
Hypertension	5906 (37.6%)	3302 (34.1%)	2604 (43.1%)
Dyslipidemia	3570 (22.7%)	1934 (20.0%)	1636 (27.1%)
Diabetes or high blood sugar	2045 (13.0%)	1152 (11.9%)	893 (14.8%)
Cancer or malignant tumor	332 (2.1%)	182 (1.9%)	150 (2.5%)
Chronic lung disease	2417 (15.4%)	1100 (11.4%)	1317 (21.8%)
Liver disease	1123 (7.1%)	519 (5.4%)	604 (10.0%)
Heart disease	3118 (19.8%)	1506 (15.6%)	1612 (26.7%)
Stroke	1000 (6.4%)	491 (5.1%)	509 (8.4%)
Kidney disease	1617 (10.3%)	714 (7.4%)	903 (14.9%)
Stomach or other digestive disease	4768 (30.3%)	2168 (22.4%)	2600 (43.0%)
Memory-related disease	484 (3.1%)	219 (2.3%)	265 (4.4%)
Asthma	893 (5.7%)	388 (4.0%)	505 (8.4%)
Emotional, nervous, or psychiatric problems	416 (2.6%)	169 (1.7%)	247 (4.1%)

Note. Values are presented as *n* (%). Participants could report more than one condition; therefore, percentages do not sum to 100%. Emotional, nervous, or psychiatric problems were retained for descriptive purposes but were excluded from the primary non-psychiatric comorbidity count.

Table S2

Comparison of sociodemographic characteristics between included and excluded age-eligible participants.

**Characteristic**	**Included participants (*n* = 15721)**	**Excluded participants (*n* = 3821)**	**Test statistic**	***p* value**	**Effect size**	**FDR-adjusted *q* value**
Age	60.90 ± 9.32	67.02 ± 11.96	$t = -29.53$	< 0.001	Cohen's d = 0.62	< 0.001
Sex			$\chi^2(1) = 49.49$	< 0.001	Cramér's V = 0.05	< 0.001
Male	7678 (48.84%)	1624 (42.50%)				
Female	8043 (51.16%)	2197 (57.50%)				
Marital status			$\chi^2(1) = 400.49$	< 0.001	Cramér's V = 0.14	< 0.001
Married/cohabiting	13763 (87.55%)	2853 (74.67%)				
Unmarried/divorced/widowed	1958 (12.45%)	968 (25.33%)				
Residence			$\chi^2(1) = 5.89$	0.015	Cramér's V = 0.02	0.015
Rural	9319 (59.28%)	2347 (61.42%)				
Urban	6402 (40.72%)	1474 (38.58%)				
Education			$\chi^2(3) = 757.51$	< 0.001	Cramér's V = 0.20	< 0.001
Less than primary school	5573 (35.45%)	2280 (59.67%)				
Primary school	4935 (31.39%)	816 (21.36%)				
Middle school	3260 (20.74%)	452 (11.83%)				
High school or above	1953 (12.42%)	273 (7.14%)				

Note. Values are presented as *mean* ± *SD* or *n* (%). Age was compared using Welch's independent-samples t-test, whereas categorical variables were compared using chi-square tests. Cohen's d and Cramér's V were used as measures of effect size for continuous and categorical variables, respectively. q values were calculated using the Benjamini–Hochberg false discovery rate procedure.

Table S3

Sensitivity and interaction analyses for the association between arthritis/rheumatism and depressive symptoms.

Panel A. Sensitivity analyses

**Analysis**	**Sample size**	**Arthritis/rheumatism *B***	**95% CI**	***p* value**
Primary complete-case model	15721	0.99	0.80, 1.19	< 0.001
Psychiatric condition included in comorbidity count	15721	0.98	0.79, 1.18	< 0.001
Reduced-adjustment model	15721	1.75	1.54, 1.95	< 0.001
Additional adjustment for general body pain severity	15721	0.54	0.35, 0.74	< 0.001
Multiple-imputation model	19542	0.93	0.75, 1.10	< 0.001

Panel B. Interaction analysis

**Term**	***B***	**95% CI**	***p* value**
Arthritis/rheumatism × comorbidity count	0.10	−0.02, 0.21	0.098

Note. B = unstandardized regression coefficient; CI = confidence interval. The primary model adjusted for age, sex, education, marital status, residence, self-rated health, nighttime sleep duration, and the 0–12 non-psychiatric comorbidity count. The psychiatric-condition-inclusive model additionally included self-reported emotional, nervous, or psychiatric problems in the comorbidity count. The reduced-adjustment model adjusted for age, sex, education, marital status, residence, and the other-chronic-condition count, but excluded self-rated health and nighttime sleep duration. The pain-adjusted model additionally included general body pain severity as a categorical variable, with “none” as the reference category.

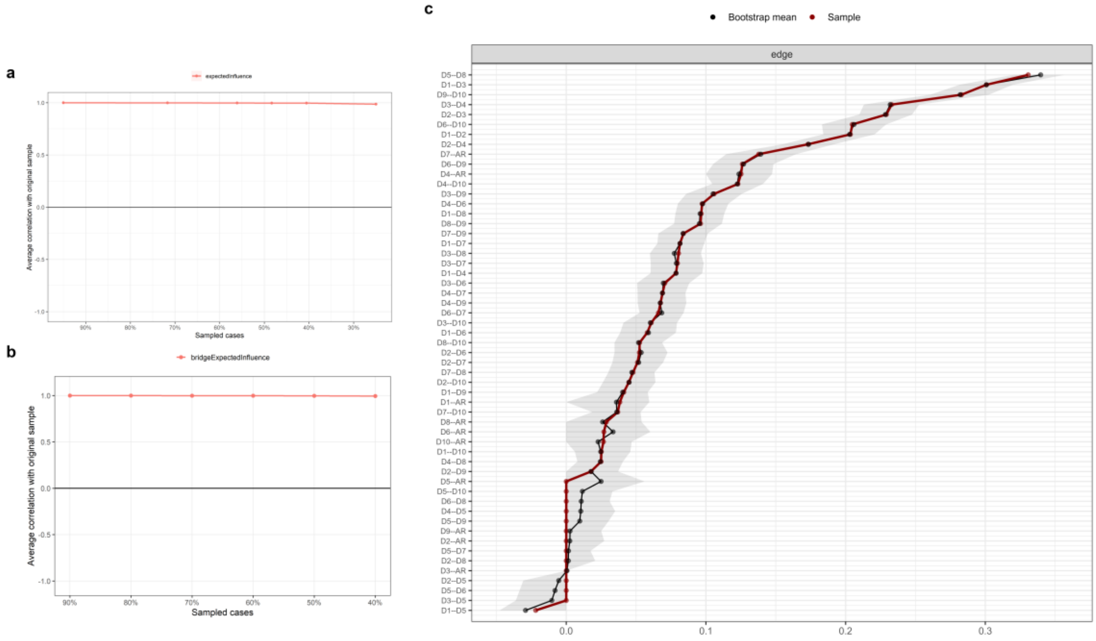

Figure S1. Bootstrap accuracy and stability of the arthritis/rheumatism–depressive symptom network. a) and b) Case-dropping bootstrap results for expected influence (EI) and bridge expected influence (BEI), respectively. c) Nonparametric bootstrap results for edge-weight accuracy, with grey areas representing 95% confidence intervals. AR = arthritis/rheumatism.

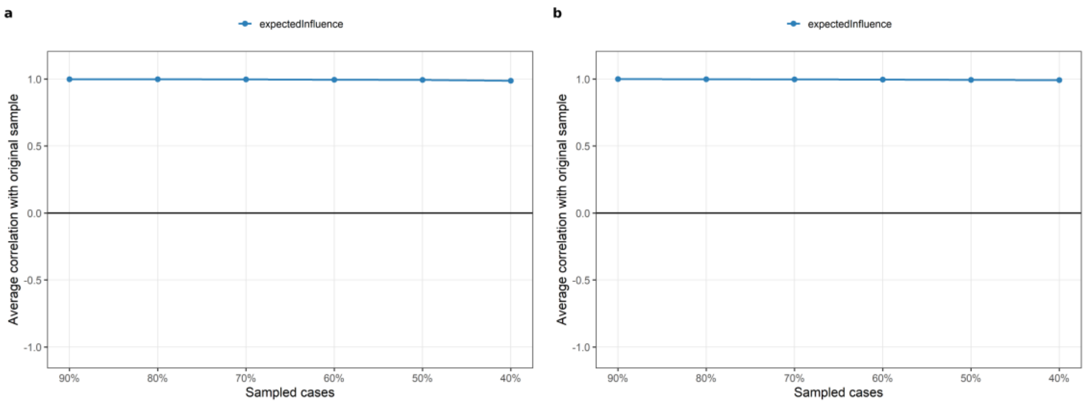

Figure S2. Case-dropping bootstrap stability of expected influence (EI) in participants with and without arthritis/rheumatism. a) Participants with arthritis/rheumatism. b) Participants without arthritis/rheumatism. Lines represent the average correlation between centrality estimates obtained from subsets of the data and those from the full sample.
